# Exposure Intimacy: A New Metric for Assessing Chemical Intake

**DOI:** 10.1289/ehp.120-a474b

**Published:** 2012-12-03

**Authors:** Kellyn S. Betts

**Affiliations:** For more than a dozen years Kellyn S. Betts has written about environmental contaminants, hazards, and technology for solving environmental problems for publications including *EHP* and *Environmental Science & Technology*.

A new way to efficiently estimate human intake of a chemical based on its production and how it is used demonstrates that people’s “exposure intimacy” with widely used chemicals varies dramatically [*EHP* 120(12):1678–1683; Nazaroff et al.]. For example, the plasticizer bisphenol A (BPA) is one of the world’s most heavily used chemicals, but its U.S. intake-to-production ratio (IPR) is over 100,000 times less than that of methyl paraben, a food preservative and antifungal agent. IPR estimates for human exposure to five commonly used phthalate plasticizers, the disinfectant *p*-dichlorobenzene, and the antibacterial agent triclosan fall in between these two extremes.

The IPR, reported as ppm, quantifies the fraction of the total amount of a chemical used in a country each year that gets into the country’s population. The metric is calculated by dividing the population’s estimated total intake of a chemical by the rate at which that chemical is produced or imported. So, for example, diethyl phthalate’s rating of 7,700 ppm indicates that for every 1 million g of the chemical entering U.S. commerce, approximately 7,700 g is taken up by the aggregate population. The metric does not reflect the distribution of exposures within the population.

For this study, intake estimates for the nine chemicals assessed were based on urinary excretion data from the U.S. Centers for Disease Control and Prevention. The chemical manufacture and import data came from the U.S. Environmental Protection Agency’s Chemical Data Reporting system.

The nine chemicals yielded a broad span of IPRs, which the researchers believe is a result of the variable opportunities for exposure associated with different chemical uses. For example, although the presence of BPA in the linings of food cans has a high potential for human exposure, its low IPR of 0.6 ppm likely reflects the fact that most BPA is used in polycarbonate plastics, where its potential for transfer to humans is relatively low. In contrast, methyl paraben’s IPR of over 180,000 ppm is consistent with its presence in products with which people have close contact—foods, cosmetics, and pharmaceuticals.

The authors acknowledge that the limited availability of biomonitoring data currently hinders widespread use of the IPR. They nonetheless foresee that the metric could contribute to a framework for rapidly estimating human exposure to untested chemicals based on the chemicals’ intended uses and anticipated production volumes.

